# A potential hidden layer of meteorites below the ice surface of Antarctica

**DOI:** 10.1038/ncomms10679

**Published:** 2016-02-16

**Authors:** G. W. Evatt, M. J. Coughlan, K. H. Joy, A. R. D. Smedley, P. J. Connolly, I. D. Abrahams

**Affiliations:** 1 School of Mathematics, University of Manchester, Manchester M13 9PL, UK; 2 The School of Earth, Atmospheric and Environmental Sciences, University of Manchester, Manchester M13 9PL, UK; 3 Centre for Atmospheric Science, The School of Earth, Atmospheric and Environmental Sciences, University of Manchester, Manchester M13 9PL, UK

## Abstract

Antarctica contains some of the most productive regions on Earth for collecting meteorites. These small areas of glacial ice are known as meteorite stranding zones, where upward-flowing ice combines with high ablation rates to concentrate large numbers of englacially transported meteorites onto their surface. However, meteorite collection data shows that iron and stony-iron meteorites are significantly under-represented from these regions as compared with all other sites on Earth. Here we explain how this discrepancy may be due to englacial solar warming, whereby meteorites a few tens of centimetres below the ice surface can be warmed up enough to cause melting of their surrounding ice and sink downwards. We show that meteorites with a high-enough thermal conductivity (for example, iron meteorites) can sink at a rate sufficient to offset the total annual upward ice transport, which may therefore permanently trap them below the ice surface and explain their absence from collection data.

## Abstract (web summary)

Collection data suggest the proportion of iron-based meteorites recovered from Antarctica is significantly lower than the rest of the world. Here, the authors propose a mechanism to explain this discrepancy, showing that iron meteorites heated by solar energy can move down through the ice, not to re-emerge.

## Graphical abstract

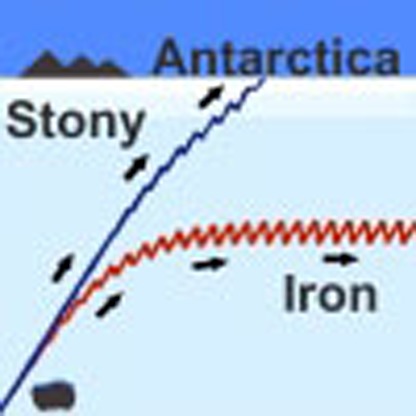

When meteorites fall onto a large area of inland Antarctica, the subsequent ice flow dynamics direct many of them to localized surface regions called meteorite stranding zones (MSZs)^1^. This concentration phenomenon (see Supplementary Note 1) allows for efficient human-led recovery missions^2^,^3^: up until December 2015, some 34,927 meteorites (official approved names of stones classified by the Meteoritical Nomenclature Committee^4^) were recovered from the surface of Antarctica, representing 66.3% of the world's total number of collected specimens. (NB there are many Antarctic stones recovered by some national collection programs that have never been formally classified and others that have only been given provisional names). However, while meteorite falls should be distributed almost uniformly across the Earth's surface, meteorite collection data (see Table 1) reveals that the proportion of iron-based meteorites (iron meteorites and stony-iron meteorites) recovered from Antarctica, 0.7%, is significantly lower than the proportion recovered after witnessed falls (see Supplementary Note 2) from the rest of the World^4^,^5^,^6^,^7^,^8^, 5.5%—a statistical difference at over the 99.9% confidence level. This comparison suggests that one or more physical mechanisms are resulting in an apparent shortfall of iron-based meteorite falls in Antarctica. Another notable statistic of the collection data comes from Antarctica's moraine-free LaPaz Icefield MSZ (86° 22′ S, 70° 0′ W), which has produced a significantly lower proportion of iron-based meteorites (0.3%) than the rest of Antarctica combined (the latter data includes debris-covered regions, glacial moraines, ice tongues and so on)—a difference at the 94% confidence level. With collection methods across Antarctica by different human searching programmes broadly similar, using visual inspection from snowmobiles (rapidly covering large areas of blue ice) or on foot (a more localized approach often focusing on rock-rich glacial moraine fields), this further suggests that the physical cause for the disparity is more pronounced in regions of debris-free MSZ ice.

It is our hypothesis that the under-representation of iron-based meteorites in Antarctic data is caused by solar energy penetrating the clear ice of the MSZs^9^. To expand: with the intensity of transmitted radiation diminishing with depth^10^, a meteorite being transported up toward the surface of a MSZ^2^,^11^ will be exposed to an increasing level of solar warming. If the rising meteorite could reach a shallow-enough depth for the solar energy to enable melting of its surrounding ice, the meteorite would sink relative to the upwelling ice. Furthermore, if the thermal properties of iron meteorites enabled them to sink faster than other meteorite classes, and counter all annual upward glacial transportation, it would provide a physical mechanism that traps iron meteorites within the ice, thus explaining their under-representation on the surface.

Under our hypothesis, sinking (relative to the upwelling ice) of englacial meteorites in an Antarctic MSZ will be seasonally dependent. In the winter months, with little-to-no solar radiation, sinking will not be possible, and so all classes of englacial meteorites can be expected to rise with the speed of the ice (whose surface is still ablating due to scouring by the strong winter katabatic winds^12^). Conversely, in the long daylight hours of the summer months, solar warming will enable susceptible meteorites to have a local rate of melting that offsets the upwelling process, and hence, for certain meteorite classes, sinking relative to the ice surface can (re)commence. The question, thus, becomes: could iron and stony-iron meteorites have a propensity to achieve a summertime-averaged relative velocity that is sufficiently large (and negative) to offset all annual upwelling, thereby permanently trapping them below the ice surface?

To answer this question, we first present the results of a series of controlled laboratory experiments that prove englacial solar warming can cause shallow-enough sub-surface meteorites to move down through the encasing ice, under the action of thawing and freezing. We then present a mathematical model of the energy balance within the system which gives a close fit to the laboratory results, allowing us to confidently apply it to an Antarctic MSZ situation. In so doing, we show how the thawing and freezing process will typically negate all annual upward transportation of a MSZ meteorite with a high-enough thermal conductivity (for example, iron), while allowing meteorites with lower conductivities to emerge from the ice. As a consequence of this filtering mechanism, the model suggests a few tens of centimetres beneath the ice surface of a MSZ, there are sub-layers of ice that potentially contain a (sparse) distribution of meteorites with high-thermal conductivities. With meteorites constantly being englacially transported towards many MSZs^13^, these layers (which are hidden from surface-searching methods) could harbour an additional reserve of iron-rich meteorites. If accessed, this layer would lead to a significant increase in our library of iron and stony-iron meteorite types, which will directly help our understanding of early solar system-formation processes and the diversity of planetesimals that were present^14^,^15^.

## Results

### Laboratory results

**Figure 1:**
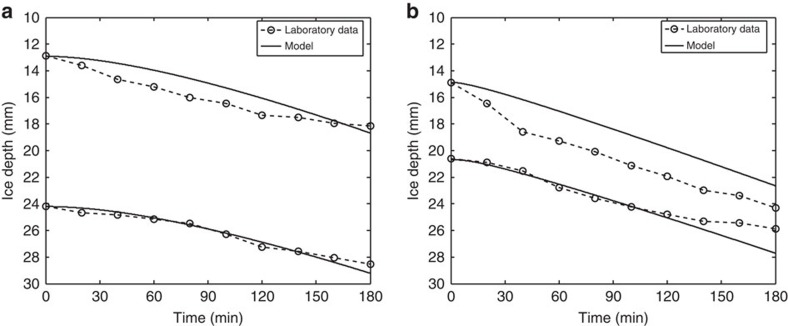
Laboratory results. Experimental results (circles and dashed line) for the upper-surface depth (relative to the ice surface) of a sinking englacial meteorite as time progresses, where the samples are exposed to solar warming from above the ice. Two sets of results for an (**a**) ordinary chondrite and (**b**) iron meteorite. These data points have a measurement error of under ±1 mm. The solid lines represent the corresponding results of our energy balance model, solved using laboratory parameter values (see the Methods section).

Our experiments centred on subjecting a meteorite encased in a block of ice to the radiation from a solar-simulator lamp held directly above the ice surface, and focused onto the meteorite (see the Methods section). Two classes of meteorite were tested: an ordinary chondrite meteorite (North West Africa (NWA) 869 L3.9–L6), and an achondritic iron meteorite (Sikhote-Alin). Both samples were of near-prolate-spheroidal geometry, with the axis of symmetry horizontally aligned with diameter 15 mm, and width 10 mm. Under the laboratory conditions, both classes of meteorite proved able to warm up enough that they could melt their surrounding ice and sink down, as shown in Fig. 1. The average speed of sinking of the iron meteorite was approximately 2.4 mm per hour, markedly higher than that for the chondrite sample at 1.5 mm per hour (although as shown in Fig. 1, their initial depths were not identical).

To relate these laboratory experiments to the Antarctic setting, we created a mathematical energy balance model that could be applied to each of the physical situations; close alignment between the model and laboratory results would give confidence to the model's Antarctic predictions. Our energy balance model necessarily considers a variety of competing energy fluxes^16^: the atmosphere's turbulent and electromagnetic heat exchanges; heat conduction within the ice, meteorite and surrounding melt-water layers; and energy to and from ice-phase transitions (see the Methods section). To run simulations, the model requires measurements of certain material properties of the meteorites: the albedo of the iron meteorite was measured as 0.159, and the chondrite meteorite as 0.106; an independent estimate of the iron meteorite's thermal conductivity was 25 W m^−1^ K^−1^ (ref. 17) and that for the chondrite meteorite was 1.5 W m^−1^ K^−1^ (ref. 18). Using these, the model's prediction for each of the experiments is overlain on the data points of Fig. 1. The closeness of fit between the experimental results and the model predictions is striking. Thus, it allows us to apply the model with confidence to the Antarctic situation, in anticipation that over the longer Antarctic time scales a more-pronounced divergence between the specimens' behaviours would be revealed.

### Antarctic results

**Figure 2:**
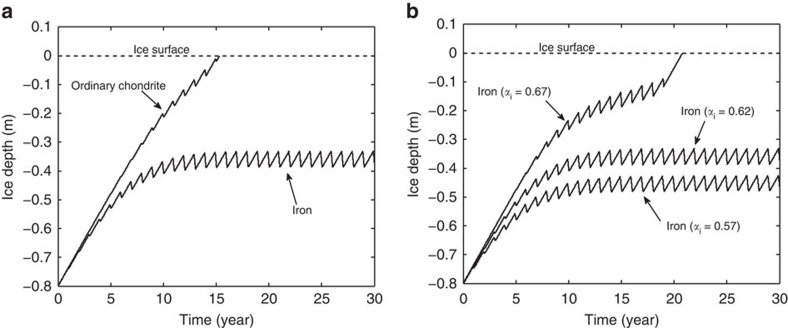
Antarctic results. Energy balance model results for the Antarctic situation, where parameter values are chosen in accordance with conditions at the Frontier Mountain Meteorite Trap area^19^. The results in **a** show the progress as time progresses of two meteorites, with thermal conductivities *k*_*m*_=1.5 W m^−1^ K^−1^ (a typical value for an ordinary chondritic meteorite) and *k*_*m*_=25 W m^−1^ K^−1^ (a typical value for an iron meteorite). In **b** the thermal conductivity is held fixed at 25 W m^−1^ K^−1^, but the ice surface albedo *α*_*i*_ is varied by ±7.5%, highlighting the sensitivity of the meteorite's progress to the reflectivity of the ice surface (and thus also the downwelling shortwave energy flux *S*_net_).

We parameterised our energy balance model to Antarctica's (much studied) Frontier Mountain meteorite trap^19^ (72° S, 160° W, see collection statistics in Table 1) and allowed the model's shortwave energy flux, longwave energy flux and sensible heat flux to vary seasonally (see the Methods section). To compare the progress between a chondrite and an iron meteorite, we used a meteorite thermal conductivity of *k*_*m*_=1.5 W m^−1^ K^−1^ for the chondrite meteorite numerical experiments^17^,^18^, and *k*_*m*_=25 W m^−1^ K^−1^ for the iron meteorite calculations^17^. A meteorite width of 3 cm and, initially, an averaged meteorite broadband (exterior) surface albedo *α*_*m*_ of 0.13 were used for both samples. Using Antarctic climatic parameters (see Table 2), results for the englacial progress of a chondrite and an iron meteorite are shown in Fig. 2a. The results clearly demonstrate the anticipated divergence between meteorite classes; over the longer Antarctic time scales a meteorite with a high-enough thermal conductivity (for example, iron) can potentially remain trapped below the ice surface (in this instance at a depth of around 35 cm), whereas a meteorite with a lower thermal conductivity (that is, iron-poor chondritic and achondritic types) will emerge onto the surface. For our particular parameter values, we found that meteorites with thermal conductivities higher than ∼4 W m^−1^ K^−1^ will remain trapped within the ice. However, given the inevitable stochastic fluctuations around our mean values, meteorites predicted to be trapped at a shallow-enough depth can sometimes still be expected to emerge from the surface. This low propensity for Frontier Mountain iron/stony-iron meteorites to reach the surface is consistent with the collection data of Table 1. The results in Fig. 2 also demonstrate that chondrites can undergo an annual freeze–thaw cycle while trapped within the uppermost half-metre of ice, which is consistent with in-ice weathering observations from englacial meteorites^20^.

The energy balance model is able to identify further meteorite parameters that might affect englacial trapping or release, for example, its broadband surface albedo and mass density. It is found that variations in meteorite surface albedo alter the predicted meteorite depths by only a modest amount: a significant ±50% change in meteorite surface albedo was found to alter the iron meteorite depth of Fig. 2a by around only ±2 cm. This small depth variation is not unexpected, as these large albedo deviations correspond to only a ±7.5% variation in absorbed solar energy; and with meteorite surface albedo unlikely to differ widely (including our measured values) and consistently between iron and chondritic meteorites (assuming the dark fusion crust is reasonably intact), we conclude that meteorite surface albedo is not the cause for the under-representation of iron-based meteorites. Likewise, meteorite mass density is not found to be a viable differentiator for englacial sinking between meteorite classes, as mass density can be shown to have a negligible impact on slow heat-transfer processes and englacial gravitational separation^6^, and any alteration in the melting point of ice due to the pressure of the overlying meteorite is minute^21^. As such we can use the model to conclude that thermal conductivity is the dominant parameter governing the divergent englacial behaviour between meteorite classes.

A notable parameter that uniformly affects the dynamics within our modelling is the ice broadband albedo *α*_*i*_ (which also acts as a direct proxy for variations in the solar flux scattered back from ice in the vicinity of the surface). So far we have used a default mean value of 0.62 based on field measurements of blue ice^12^. Yet this parameter varies depending on the optical quality of the local ice (for example, blue ice versus white ice versus snow-covered ice). In Fig. 2b we show how our results for the iron meteorite are altered when *α*_*i*_ is varied by ±7.5% (equivalent to a ±12% variation in downwelling solar radiation): the higher albedo is now sufficient for the iron meteorite to emerge, whereas the lower albedo deepens the meteorite's resting depth by some 10 cm. As this result suggests, a light covering of snow (or shading by local topography) on a MSZ could allow iron-based meteorites to emerge, helping explain why a small number of iron-based meteorites are still found on the surface of Antarctica's MSZs, and trapped in glacial moraine fields. Conversely, it is possible to adjust the model parameters (for example, to simulate periods of higher average summer insolation) so as to have the chondritic meteorites also permanently englacially trapped. Interestingly, blue-ice fields in Greenland of similar latitude and elevation, but with a higher average temperature profile than Antarctica, have not yielded any surface meteorites, consistent with our work and aligning with previous suggestions for their absence^22^.

Finally, we found the intuitive result that larger meteorites are transported to the ice surface more readily then smaller ones—this being due to the ability of smaller meteorites to more efficiently transmit heat internally to the underlying ice for melting. We observed a near-linear dependence of the englacial resting depth against the meteorite width *w*, where iron meteorites of widths >22 cm emerged at the ice surface (all other parameters as given in Table 2). This result, of course, assumes the meteorite does not break up during the englacial freeze/thaw process. This is an important caveat to make, for the high number of freeze/thaw cycles an iron meteorite can be expected to go through (see Fig. 2) may explain why smaller masses of iron meteorites are recovered from Antarctica as compared with the rest of the world^2^.

## Discussion

The plausible implication of these results is the existence of a sparsely distributed layer of iron-based meteorites underneath the surface of Antarctica's MSZs. With collection methodologies from MSZs currently based on visual recognition at the ice surface^2^, any attempt to determine if such a layer exists would require a significant change in detection and collection strategies. Furthermore, any such collection approach must be able to easily cover relatively large areas of ice. To highlight why, one can use existing collection data to infer a rough number density of iron-based meteorites within a particular MSZ. For the debris-free LaPaz Icefield (Table 1, where we assume that the meteorite fall data to accurately represent the proportion of meteorites recovered: see Supplementary Note 2), some 92 iron-based meteorites appear ‘missing' from the searched area of roughly 100 km^2^. This allows us to crudely estimate the LaPaz number density of distributed englacial iron meteorites at around one per square kilometre. Yet the near absence of terrestrial rock from debris-free MSZs, means the number of false positives that would be detected in a meteorite recovery mission would be negligible. So even though the density of missing iron meteorites is low, suitably focused meteorite collection programmes from debris-free areas of MSZs, may well be feasible.

Along with the value of answering an outstanding scientific question, the motivations for accessing such a layer are clear: every new iron or stony-iron meteorite sample recovered has the potential to have originated from the core^23^ (or core–mantle boundary) of its own unique parent asteroid body providing insights into the number, diversity, evolution^24^ and destruction^15^,^25^,^26^ of protoplanets that existed in the early solar system. This knowledge would fill critical gaps in our understanding of both how different meteorite groups are related to one another^27^, and the chemical heterogeneity of the solar nebula^24^,^28^,^29^, from which these bodies were accreted and differentiated.

## Methods

### Energy balance model

Our energy balance model is designed to capture the essential physics underpinning the sinking process of an englacial meteorite while avoiding extraneous details. The model does not attempt to offer highly accurate Antarctic site-specific results; it aims to address the proof-of-concept question of englacial sinking through the inclusion of dominant (or aggregated) terms in the various energy balance equations and so should be taken to offer generalized quantitative predictive results at this stage.

We consider three distinct stages within our modelling. Chronologically, the evolution starts with the meteorite heating due to absorption of downwelling solar radiation through the ice; however, it remains fully encased in ice while temperatures remain below freezing throughout. Then, once the top surface of the meteorite is sufficiently heated it is able, through conduction, to induce melting of the overlying ice, allowing an upper water layer to form; this water layer can itself evolve through its interaction with both the overlying ice and the underlying meteorite. Next, if the meteorite continues to warm sufficiently, melting at its lower surface will also commence. When it does, we allow the sub-meteorite melt water to be squeezed upwards by the weight of the meteorite adding to the overlying water layer, thereby enabling the meteorite to sink downwards and fill the displaced volume. These three stages can stop at any time, and freezing reoccur, as the meteorological inputs vary, thus bringing the sinking process to a halt (this being the case for the Antarctic situation during the winter months).

**Figure 3:**
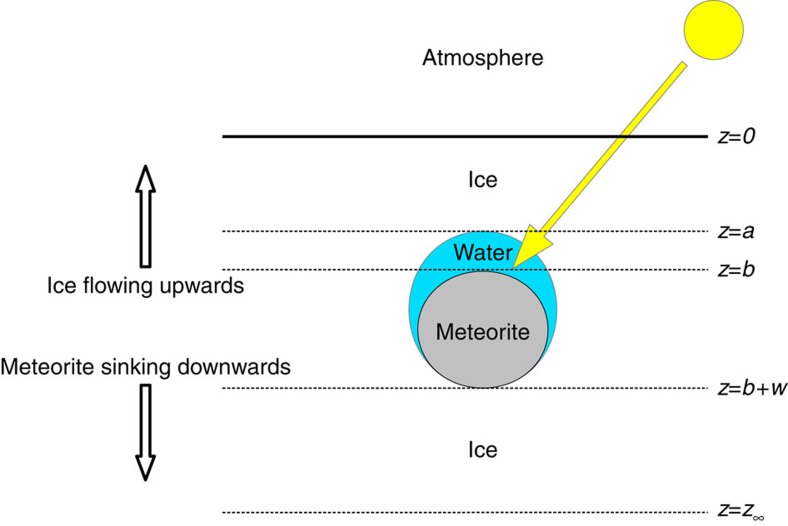
Model geometry. A (not to scale) schematic diagram highlighting the boundaries and geometry of the mathematical model for the Antarctic situation, in which an englacial meteorite is exposed to solar radiation.

To model this process in the simplest and most elucidating manner, we consider a one-dimensional representation to the physical problem, as sketched in Fig. 3. Thus, the model has four distinct regions at increasing depth, *z*: (i) an upper ice layer that is exposed to the atmosphere; (ii) a water layer (which need not always exist); (iii) the meteorite; and (iv) a lower ice layer. To model the Antarctic situation, one must incorporate the relative motion of the meteorite to the ice surface. With the MSZ surface in equilibrium at *z*=0, say, the upwelling ice velocity *V* must be matched by the ablation rate (which is the sum of the energetic sublimation rate *v*, and the non-energetic rate that ice is scoured off the surface by the katabatic winds, *V*−*v*).

To frame the problem mathematically, we need to consider the energy balance at the five boundaries/interfaces of the four regions. It is of note that two of these interfaces are ‘free-boundaries', whose locations are variables that require solving as part of the problem: the upper ice/water interface (*z*=*a*), and the water/meteorite interface (*z*=*b*). The time-varying solution to these two variables thus determines the dynamics of the meteorite within the ice. In solving for them, we must also solve for the other variables involved within the model, namely the temperatures *T*_*j*_ in regions *j*=1−4 (note that the air temperature is a model input).

At the atmosphere/ice interface (*z*=0), the energy balance is given by^16^:


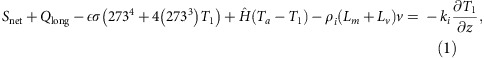


where, sequentially, the terms on the left hand side represent: the contribution of the shortwave solar flux; the incoming longwave flux; the linearized outgoing longwave radiation (Stefan–Boltzmann's law); the sensible heat flux; and sublimation, respectively. The right hand side is the heat flux into the ice, which is given by Fourier's heat-transfer law. All model parameters are defined in Table 2.

At the ice/water interface (*z*=*a*; once it exists) the temperature must be at 0 °C. Further, the energy balance tells us that the energy flux for melting the ice (or its refreezing) plus the heat flux into the ice must equate with the heat flux from the water layer:


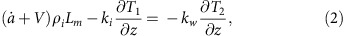


where the overdot denotes differentiation with respect to time. Note that the inclusion of +*V* on the left hand side is due to the frame being fixed with respect to the upwelling ice. When the water layer does not exist, we neglect the phase change term in (2) and *k*_*w*_ becomes *k*_*i*_.

At the water/meteorite interface (*z*=*b*), we must consider the shortwave solar energy that is reaching the meteorite surface (the longwave flux having been absorbed at the surface), and how this flux decays with depth below the surface. To achieve this we make use of the Beer–Lambert's exponential decay law with extinction coefficient *γ*_*i*_. The balance at this interface is given by the solar energy flux plus the heat flux from water layer equating with the heat flux into the meteorite:


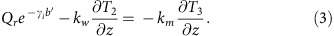


Here *Q*_*r*_ may be thought of as the shortwave energy that would be absorbed by the meteorite in the absence of absorption/scattering by the ice, and thus *Q*_*r*_=(1−*α*_*i*_)(1−*α*_*m*_)(1−*s*_*h*_)*S*, where *S* is the total incoming solar flux, *α*_*i*_ is the ice surface broadband albedo, *α*_*m*_ is the broadband meteorite albedo and *s*_*h*_ is the percentage of incoming solar radiation that is shaded (thus capturing the effects of local topography). Due to the inclination of the sun to the horizontal (solar elevation angle *θ*), we make use of an effective distance *b*′ that represents the distance travelled while being attenuated within the ice and melt water (where we neglect the tiny effect of refraction within the very-thin melt-water layer):


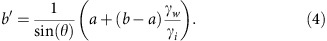


At the lower meteorite/ice interface (*z*=*b*+*w*), where *w* is the meteorite width, one needs to compute whether the temperature is high enough to melt the ice. When the interface temperature is at 0 °C, melting is permitted, and the energy balance is given by the heat flux from the meteorite layer equating with the energy flux for melting plus the heat flux into the lower ice layer:


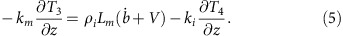


Again, the +*V* term is added due to the moving frame of reference. When the temperature is below melting point, we neglect the phase change term in (5).

At the bottom of the lower ice region we shall prescribe a temperature-gradient condition of the form


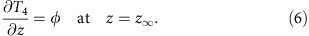


In the laboratory, where the ice block is relatively warm and thin (5 cm), and the incoming heat fluxes held constant, we use a measured value of the ice temperature *T*_∞_ at *z*_∞_. Thus, the ‘far-field' temperature gradient is


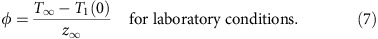


However, in the Antarctic setting we are unable to prescribe such a measured temperature at *z*_∞_, as the temperature profile within the ice varies with time (albeit slowly). As an alternative, we note that the ice underneath the meteorite must tend to the temperature profile of the surrounding ice, and so we must prescribe a condition drawn from the meteorite-free situation, that is, we need to match the temprature flux at *z*_∞_ with that of the ice thermal boundary layer. To achieve this, we can utilize the fact that the magnitude of the (meteorite-free) annual ice-temperature variation in the boundary layer diminishes rapidly with depth, which means that in practice there is only a small average annual temperature gradient at *z*_∞_ (ref. 30). To the order of accuracy of other assumptions made in this paper, it is reasonable to assume that *z*_∞_ is sufficiently deep so that the temperature gradient can be taken as zero there; hence we take


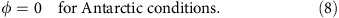


As a robustness check, we computed results for small variations from *ϕ*=0, and found only minor quantitative differences between them, thus showing that this is a reasonable condition to impose.

To prescribe the underlying equations within each region, we need to consider the conservation of heat energy. While one could use the full heat equation (*ρcT*_*t*_=*kT*_*zz*_, where *c* is the specific heat capacity of the medium in question), we are able to simplify matters by considering the time scales involved. By noting that the critical depth scale in the model is the annual ice uplift height *H* (*H*=*V* × 1 yr), it allows us to compute the associated time scale of sinking Λ_1_, as ∼10 days (for the Antarctic parameters given in Table 2). The latter is found by comparing the heat flux required for sinking the meteorite 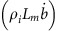 with the solar forcing felt on it 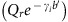, and so 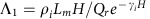. In contrast, the time scale Λ_2_ for which the full heat equation will relax to its steady-state version (*T*_*zz*_≈0), can be shown to be the order of 1 h (Λ_2_=*ρ*_*i*_*cH*^2^/*k*_*i*_). With this large relative difference between time scales 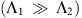, we need only consider the steady-state version within each region *j*=1–4 (where we are implicitly assuming *z*_∞_ is suitably shallow for the steady-state heat equation to be used, and simultaneously deep enough for the annual temperature variation to be minor), namely:


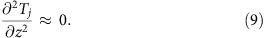


It is of note that time dynamics are still present within this model, via the annual variation in incoming solar radiation, thereby making our model quasi-steady. In addition, the steady-state heat equation coupled with the prescription of *ϕ*=0 for the Antarctic situation, sets the ice-temperature within region 4 as that of the meteorite base. This removes any dominating heat flux within region 4, making results computable from regions 1–3 only, and so an exact position for *z*_∞_ is not required in that instance.

Rearrangement of the above equations yields a numerically tractable set of non-linear differential equations for *a* and *b* in terms of the input parameters. The particular form of their solutions depends on which stage of the sinking process is currently in effect. During the first stage, when the meteorite is encased in ice and moving with the upwelling ice, the speed of the interfaces *a* and *b* will be −*V* and the surface temperature of the meteorite will be below zero. In this case, calculation of the linear temperature profile is straightforward from equations (1, 2, 3, 4, 5, 6, 7), with no time evolution of *a* and *b* to solve for.

During the second stage, where melting of the lower ice has not yet commenced but the upper water layer is in existence (*b*−*a*>0), one can compute the location of the ice/water interface, *a*, from the dynamic equation


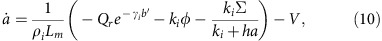


where


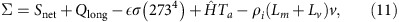



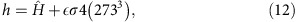



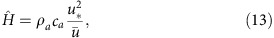



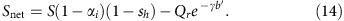


This last term in *S*_net_ is necessary to ensure conservation of total solar radiation. Within this relation we are (in effect) assuming that any scattered shortwave energy within the ice only affects the ice at the atmospheric interface, that is, the shortwave energy directly warms only the ice surface and the meteorite (which then heats up the remaining ice by conduction). This assumption, which greatly simplifies the analysis, is expected to lower very slightly the temperature of the ice near the meteorite compared with reality. This consequently reduces the rate of sinking, and thus is, by design, a slightly conservative estimate (note that the numerical experiment confirms that, even when the last term in *S*_net_ is removed completely, there is still little quantitative change to the results).

Once melting of the lower meteorite/ice surface (*z*=*b*+*w*) has commenced, and thus the meteorite starts to sink, one must switch to computing the coupled pair of equations,


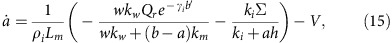



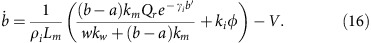


To determine whether or not melting of the lower surface has begun, one can simply check whether or not the velocity of *b* in (14) is positive: while it is positive, we solve for *a* and *b* from (13) and (14), and if its numerical value ever becomes negative (no melting), one takes *b* as fixed and reverts to determining the evolution of *a* from equation (8).

**Figure 4:**
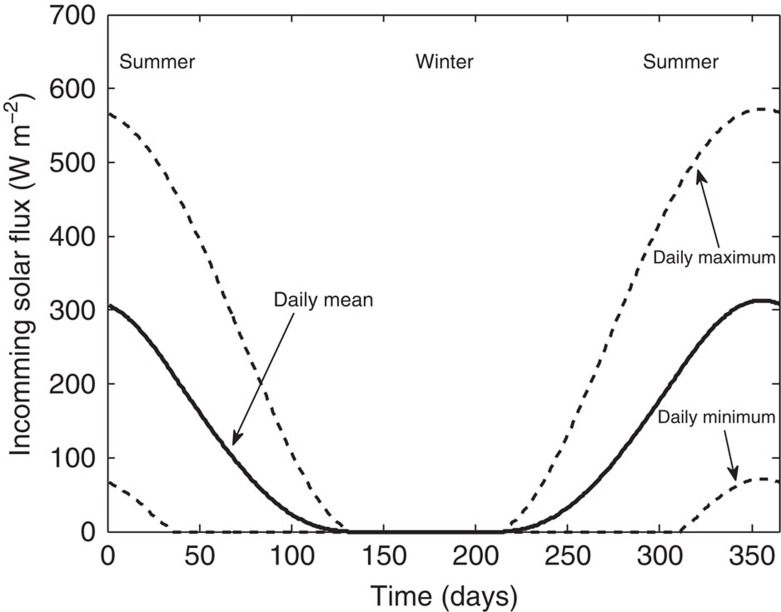
Ice surface solar energy. The computed incoming shortwave energy flux S(t) reaching the Frontier Mountain meteorite trap ice surface, showing the daily mean (solid line), maximum daily value (upper dashed line) and minimum daily value (lower dashed line). These were calculated using the libRadtran atmospheric radiative-transfer model^31^ (as detailed in the Methods section).

The final aspect to note in the Antarctic representation of the model is that the atmospheric energy fluxes are seasonally dependent. To account for this we calculated and used the six-hourly average shortwave flux *S*(*t*) from the libRadtran atmospheric radiative-transfer model^31^, which incorporated a pseudo-spherical approximation with parameter inputs made relevant to the (relatively) well-parameterised Frontier Mountain Meteorite Trap area climatology^32^,^33^,^34^,^35^,^36^ (see Table 1 for details of meteorite collection in this locality). The period of this time average was chosen so as to maintain as much diurnal granularity as possible, without violating our assumption of a quasi-steady heat model, that is, 6 h
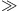
Λ_2_≈1 h (although results for durations between 1 h and 12 h showed only a minor quantitative difference). The computed maximum, minimum and mean daily values for the incoming shortwave solar flux *S* are shown in Fig. 4, where the diurnal variations are sinusoidal (and so the six-hourly averages lie within the shown range). To reflect the fact that the seasonality in shortwave energy has a direct effect on air temperature *T*_*a*_ and thus the incoming longwave energy flux *Q*_long_, we allow these parameters to also be seasonally dependent^37^. To achieve this in a straightforward manner, we use a normalized version of the six-hourly shortwave profile, denoted by 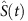 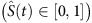, to approximate their dynamics, namely:


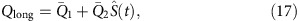



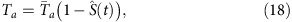


where 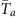 is a negative constant measured in °C (thus the maximum air temperature is attained during summer and the minimum temperature during winter), and 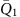 and 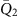 are both positive constants. With this model formulation, we are able to solve for the Antarctic situation, as well as the laboratory case (where *θ*=90° and the incoming energy fluxes are held constant).

The parameter values used in the laboratory simulation and the Antarctic simulation are all stated in Table 2. The *in situ* Antarctic parameter values are all in relation to the Frontier Mountain Meteorite Trap area^19^. Suitable proxies were taken for the blue-ice thermal conductivity *k*_*i*_ (ref. 38), the surface roughness estimate u* ref. 30 and the solar attenuation parameter through water *γ*_*w*_ (ref. 39) and ice *γ*_*I*_ (refs 12, 39). When a range of parameters were provided, we used an averaged value. The percentage incoming solar radiation that is shaded, *s*_*h*_, is a time-averaged value inferred from the neighbouring mountain elevations^17^. The six-hourly solar elevation angles *θ* were computed^40^, while in the laboratory the solar-simulator lamp was held directly overhead (that is, 90°). The meteorite width *w* used in the Antarctic situation was chosen so as to be indicative of typical collected Antarctic meteorite specimens^2^ (although these smaller collected sizes/masses may be the consequence of larger material that broke up on the surface through repeated freeze–thaw cycles and wind action^2^). The scaling of the Antarctic longwave radiation was taken to be consistent with seasonal observations^37^. The thermal conductivity of the IIAB iron (Sikhote-Alin) was inferred from the IAB iron meteorite Campo del Cielo^17^; ±25% variations to this reasonable estimate still yielded results close to the data points (which was to be expected over the shorter laboratory time scales). The meteorite surface albedos (fusion crust cover) were independently measured for this study (see the Acknowledgements section).

The results were computed using a code written in Matlab, which the authors can supply on request. Potential extensions to our modelling approach are discussed in Supplementary Note 3.

### Laboratory experiments

**Figure 5:**
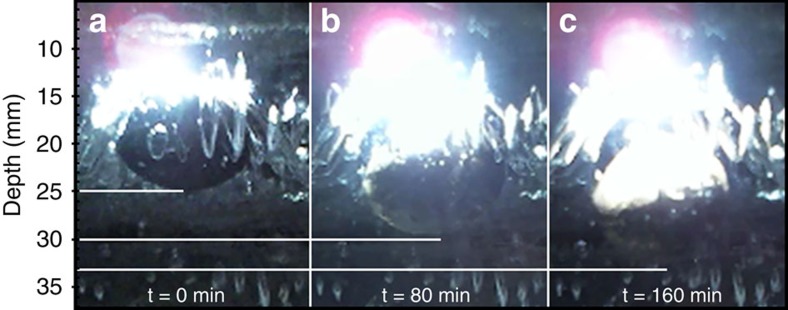
Laboratory stills. Laboratory measurements of an iron meteorite sinking through ice as discussed in the Methods section. Depth (*z*-axis) in mm is taken relative to the upper ice surface. Shown are three side-view images of the meteorite sinking downwards, taken at 80-min intervals. The images relate to the first data point (0 min), fifth data point (80 min) and ninth data point (160 min) of Fig. 1 (right panel), of the meteorite starting from 14.9 mm encapsulation depth in the ice. To align these images with the results of Fig. 1 (which show the depth of the upper meteorite surface) one must subtract the meteorite width of 10 mm from the depth of the meteorite base, which are indicated by the white lines (the location of the base is easier to observe, due to the reduced amount of glare from the light source).

Our experiments centred around subjecting a meteorite encased in a block of ice (400 × 400 × 50 mm) to the radiation from an Oriel Solar Simulator arc lamp (irradiance 1,440 W m^−2^) that was held at a constant 10 mm above the ice surface and focused onto the englacial meteorite. This was conducted in a (otherwise dark, non-reflecting) temperature-controlled room, with ambient temperature −1 °C. The meteorite movement was recorded over a 3-h period using an HD time-lapse camera positioned at the side of the ice block, from which the meteorite's progress could be measured (with an error of under ±1 mm), see Fig. 5, and to confirm that the outward-facing ice surfaces were not melting. With this experimental set-up, we were able to successfully conduct four controlled laboratory experiments, as shown in Fig. 1.

To create the blocks of ice with a meteorite included, we first slowly froze the lower half the block in a container. We then placed a meteorite on top and carefully added an upper layer of cold water, which we then slowly froze. Once frozen, we removed the container. This whole process could take 2–3 days, so as to reduce the number of air bubbles within the ice. It also helped fully bond the meteorite to its surrounding ice.

Further to our four main experiments, controlled experiments were performed to show that the meteorite only sank in the presence of the solar simulator. We also recorded temperatures within the ice to confirm that when the meteorite was sinking its base was at 0 °C, thus confirming the assumptions within our energy balance model.

## Additional information

**How to cite this article:** Evatt, G. W. *et al*. A potential hidden layer of meteorites below the ice surface of Antarctica. *Nat. Commun.* 7:10679 doi: 10.1038/ncomms10679 (2016).

## Supplementary Material

Supplementary Information Supplementary Notes 1-3 and Supplementary References

## Figures and Tables

**Table 1:** Meteorite collection statistics.

Meteorite class	Iron and stony-iron	Others
LaPaz Icefield MSZ finds (Antarctica)	5 (0.3%)	1,665 (99.7%)
Frontier Mountains MSZ finds (Antarctica)	0 (0.0%)	798 (100.0%)
Total Antarctic Finds (all search programmes)	239 (0.7%)	34,688 (99.3%)
Rest of world falls	60 (5.5%)	1,037 (94.5%)
Rest of world finds (excluding falls)	1,145 (6.9%)	15,505 (93.1%)

MSZ, meteorite stranding zone.

Statistics of classified named meteorite stones including the number and percentage of iron-based meteorite finds from the LaPaz Icefield MSZ Antarctica; Frontier Mountain MSZ Antarctica; the whole of Antarctica; the number of observed (and then collected) meteorite ‘falls' from the world excluding Antarctica; and the number of meteorite ‘finds' from the world excluding Antarctica. Data is taken from the Meteoritical Society bulletin of classified and named meteorite samples^4^, updated as of 18 December 2015. We note that this official data set does not include named meteorites with only provisional or undocumented meteorites names. Iron meteorites include all iron groups. Stony-iron meteorites include pallasites and mesosiderite types.

**Table 2:** Energy balance model parameter values.

Parameter	Description	Laboratory value	Antarctic value
*k*_*i*_	Thermal conductivity of ice (W K^−1^ m^−1^)	2.22	2.07
*k*_*w*_	Thermal conductivity of water (W K^−1^ m^−1^)	0.58	0.58
*k*_*m*_	Thermal conductivity of meteorite (W K^−1^ m^−1^)	1.5, 25	1.5, 25
*c*_*a*_	Heat capacity of air (J kg^−1^ K^−1^)	1,005	1,005
*ρ*_*a*_	Air density (J kg^−1^ K^−1^)	1.29	0.95
*ρ*_*i*_	Density of ice (kg m^−3^)	916.2	916.2
*L*_*m*_	Latent heat of melting ice (J kg^−1^)	3.34 × 10^5^	3.34 × 10^5^
*L*_*v*_	Latent heat vapourization, water (J kg^−1^)	22.6 × 10^5^	22.6 × 10^5^
*γ*_*i*_	Attenuation coefficient of blue ice (m^−1^)	2.5	2.5
*γ*_*w*_	Attenuation coefficient of water (m^−1^)	0.001	0.001
*V*	Ice sheet heave velocity (metres per year)	—	0.065
*v*	Ice sheet sublimation rate (metres per year)	Negligible	*V*/2
*α*_*i*_	Blue-ice albedo (−)	0.62	0.62
*α*_*m*_	Meteorite exterior-surface albedo (−)	0.106–0.159	0.13
*σ*	Stefan–Boltzmann's constant (W m^−2^ K^−2^)	5.667 × 10^−8^	5.667 × 10^−8^
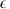	Emmissivity of ice (−)	0.94	0.94
*θ*	Solar elevation angle (°)	90	Computed
*T*_*a*_	Air temperature (°C)	−1	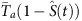
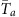	Lowest air temperature (°C)	—	−40
*S*_net_	Incoming shortwave energy (W m^−2^)	1,440	Fig. 4
*Q*_long_	Incident longwave radiation (W m^−2^)	300	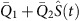
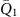	Longwave energy parameter (W m^−2^)	—	93
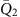	Longwave energy parameter (W m^−2^)	—	47.5
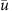	Average wind speed (m s^−1^)	2	11
u_*_	Friction velocity (m s^−1^)	0.1	0.1
*T*_∞_	Ice temperature at bottom (°C)	−4	—
*z*_∞_	Ice depth (m)	0.05	—
*ϕ*	Heat flux in region 4 (W m^−2^)	—	0
*s*_*h*_	Solar shading	0	7.5%
*w*	Meteorite width (m)	0.01	0.03

Parameter values used in our energy balance model, for both the laboratory study and the Antarctic analogy (based on the Frontier Mountain meteorite trap area; see the Methods section).
