# Supplementary material for: A potential hidden layer of meteorites below the ice surface of Antarctica
Source: Nat Commun. 2016 Feb 16;7:10679. doi: 10.1038/ncomms10679 (PMC4757754; doi:10.1038/ncomms10679)
Supplement: Supplementary Information — Supplementary Notes 1-3 and Supplementary References [file ncomms10679-s1.pdf]

Supplementary Note 1. Meteorite Concentration Mechanisms

Meteorites are recovered from all over the surface of the Earth, from hot deserts recovered by professionals and research meteorite collection trips, to serendipitous discoveries in urban areas, and areas around known ancient impact sites. In Antarctica, meteorites arrive at their collection locality via a range of scenarios including: where the meteorites originally fell; those slowly transported to MSZs via ice flow paths (the focus of this study, where we address the transport mechanisms in the final tens of centimetres below the ice surface); and those that have been transported across MSZ surfaces by high energy katabatic winds (note these samples are typically small, < 0.1 kg[1], and recovered from firn located at the ice-field edge downwind of the prevailing wind direction). In some instances, their transportation to a collection locality can be due to a combination of such factors[2,3].

Supplementary Note 2. Meteorite Collection Biases

In this study we compare the statistics of Antarctic meteorites to rest of the world meteorite fall data instead of meteorite find data, because the find data is known to be prone to collection bias, largely due to the relative ease of detecting iron-rich meteorites using metal detector search techniques (e.g. in the hot deserts of America and Oman by private meteorite hunting expeditions, compared with the challenge of identifying stony meteorites, which are often similar in appearance to terrestrial rock[4,5,6]) and the greater survivability of iron meteorites over stony ones[7]. It is, thus, reasonable to consider that the rest-of-the-world find data might over estimate the proportion of iron meteorites, and Antarctica's collection data to under estimate this proportion. However, even meteorite fall data is not necessarily immune from such biases. For example, meteorites falls only represent about 250 years of accumulation time compared with an accumulation period of tens to hundreds of thousands of years for Antarctica[4]. Therefore, collection statistics may include changes in meteorite populations through time[5,6].

To help remove all of these potential biases from the collection data analysis, one could directly test our findings and seek the missing Antarctic englacial samples from a MSZ. Clearly such a task poses some fieldwork challenges. Yet once retrieved, these samples, plus those already collected from the surface of the MSZ, should give an accurate representation of the proportions of meteorite classes reaching the Earth's surface throughout the last million years.

Supplementary Note 3. Model Geometry

Our energy balance model has significant strength in its simplicity, allowing the gross effects governing the progress of an englacial meteorite to be discerned. However, due to its simplified one-dimensional form, the model was not designed to analyse the effects of meteorite geometry. As such, the model will not pick out dynamics that depend strongly upon, say, curvature (such as studying the progress of an englacial dust particle) or a meteorite with highly non-uniform geometry. Whilst computationally-heavy mathematical approaches will be required to study these effects, we deliberately chose not to become overly engrossed in asymmetric random

geometric details (after all, no meteorite is unique). Rather, the purpose of our model was, in effect, to study the progress of a `typical' meteorite, and we leave the study of bespoke geometric aspects to future investigations.

Supplementary References

1. Harvey, R.P. Moving Targets: The effect of supply, wind movement, and search losses on Antarctic meteorite size distributions. In Workshop on Meteorites from Cold and Hot Deserts (Schultz L., Annexstad J.O. and Zolensky M.E., eds.). *Lunar and Planetary Institute Technical Report* **95**, 34–36 (1995).

2. Gow, A.J. & Cassidy, W.A. The emerging meteorite: Crystalline structure of the enclosing ice. *Smithsonian Contrib. Earth Sci.* **28**, 87–91 (1989).

3. Krahenbuhl U. & Langenauer, M. ALH 82102: An Antarctic meteorite embedded partly in ice. *Meteoritics* ***29,*** 651–653 (1994).

4. Corrigan, C., *et al.* A Statistical look at the US Antarctica Meteorite Collection. Righter, K., et al. (eds). 35 Seasons of U.S. Antarctic Meteorites (1976–2010): A Pictorial Guide to the Collection. John Wiley & Sons (2014).

5. Beven, A.W.R., *et al.* Meteorite flux on the Nullabor Region, Australia. In Meteorites: flux with time and impact effects. Editor Grady M. The Geological Society (1998).

6. Cassidy W.A. & Harvey R.P. Are there real differences between Antarctic finds and modern falls meteorites? *Geochim. Cosmochim. Acta* ***55,*** 99-104 (1991).

7. Cassidy, W. A. Meteorites, Ice and Antarctica: a personal account. *Antarct. Sci.* **16**, 87–88 (2004).
